# Studies Progression on the Function of Autophagy in Viral Infection

**DOI:** 10.3389/fcell.2021.772965

**Published:** 2021-12-16

**Authors:** Weizheng Liang, Huimin Liu, Junli He, Lisha Ai, Qingxue Meng, Weiwen Zhang, Chengwei Yu, Hao Wang, Hui Liu

**Affiliations:** ^1^ Harbin Institute of Technology, Harbin, China; ^2^ Department of Infectious Diseases, Southwest Hospital, Third Military Medical University (Army Medical University), Chongqing, China; ^3^ Department of Pediatrics, Shenzhen University General Hospital, Shenzhen, China; ^4^ Department of Teaching and Research, Shenzhen University General Hospital, Shenzhen, China; ^5^ Department of Science, Southern University of Science and Technology, Shenzhen, China; ^6^ Department of Gynaecology and Obstetrics, Shenzhen University General Hospital, Shenzhen, China; ^7^ School of Future Technology, University of Chinese Academy of Sciences, Beijing, China; ^8^ CAS Key Laboratory of Genome Sciences and Information, Beijing Institute of Genomics, Chinese Academy of Sciences, Beijing, China; ^9^ Department of Hepatobiliary Surgery, Shenzhen University General Hospital, Shenzhen, China

**Keywords:** autophagy, coronavirus, virus, infection, SARS-CoV-2

## Abstract

Autophagy is a conservative lysosomal catabolic pathway commonly seen in eukaryotic cells. It breaks down proteins and organelles by forming a two-layer membrane structure of autophagosomes and circulating substances and maintaining homeostasis. Autophagy can play a dual role in viral infection and serve either as a pro-viral factor or an antiviral defense element dependent on the virus replication cycle. Recent studies have suggested the complicated and multidirectional role of autophagy in the process of virus infection. On the one hand, autophagy can orchestrate immunity to curtail infection. On the other hand, some viruses have evolved strategies to evade autophagy degradation, facilitating their replication. In this review, we summarize recent progress of the interaction between autophagy and viral infection. Furthermore, we highlight the link between autophagy and SARS-CoV-2, which is expected to guide the development of effective antiviral treatments against infectious diseases.

## Introduction

Autophagy is an evolutionary conserved lysosome-dependent cellular metabolic pathway in eukaryotic cells by degrading senescent organelles and long-lived proteins into small molecules such as peptides or amino acids through which the material recycling and intracellular homeostasis can be maintained (Chiang, Terlecky et al., 1989; Rubinsztein, Marino et al., 2011; Wei, Liu et al., 2018; Levine and Kroemer 2019). Meanwhile, autophagy has critical functions in cell growth and development (Levine, Mizushima et al., 2011; Rubinsztein, Marino et al., 2011; Mizushima 2018). The existing studies have demonstrated that various diseases can cause autophagy dysfunction, including tumors, neurodegenerative diseases, cardiovascular diseases, and viral infections (Levine and Kroemer 2008; Mizushima 2018; Mizushima and Levine 2020). As part of the human body’s protective and defensive mechanisms, autophagy is essential in the face of pathogenic microbial invasion, whereby cells can attack the “invaders” through autophagic degradation. However, lots of pathogenic microorganisms, especially viruses, have evolved various strategies to evade host cell autophagy and infect the organism persistently, and some viruses can even use the autophagic machinery to promote their replication in the host (Choi, Bowman et al., 2018; Yang and Klionsky 2020).

The functions of cellular autophagy in viral infection have been studied elsewhere. This review focuses on a detailed portrait of the connection between autophagy and viral infections in cells. It could be useful to understand viral infection diseases and find proper medical strategies to treat them.

## Classification and Process of Autophagy

**FIGURE 1:**
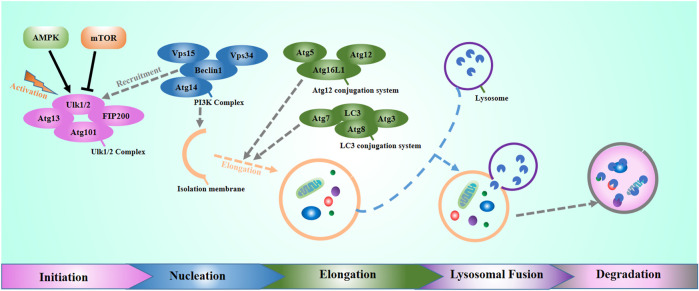
The molecular process of autophagy, including initiation, nucleation, elongation, lysosomal fusion, and degradation.

Autophagy is a ubiquitous biological mechanism in eukaryotes that contributes to maintaining homeostasis in the organism. Generally speaking, autophagy includes three main types: microautophagy, molecular chaperone-mediated autophagy, and macroautophagy (Oku and Sakai 2018). This review focuses on macroautophagy, which is the most important autophagy form. In normal physiological conditions, the intracellular autophagy level is at the basal level. The intracellular or extracellular stress can strongly bring up autophagy levels to maintain cellular homeostasis. Until now, more than 30 genes have been found to participate in the regulation of autophagy (Choi, Ryter et al., 2013; Saha, Panigrahi et al., 2018). Those genes are autophagy-related genes. In mammals, the classical autophagic pathway has five stages: initiation, nucleation, elongation, lysosomal fusion, and degradation of autophagic contents (Figure 1). Autophagy initiates at the activation of the Unc-51 like autophagy activating kinase 1(ULK1) complex. ULK1 is a mammalian homolog of autophagy-related gene 1 (ATG1), which interacts with the adherent plaque kinase family (FAK family), the autophagy-related gene Atg13, and Atg101 to form the ULK1 complex (Zachari and Ganley 2017). ULK1 activation promotes the recruitment of multiprotein complexes with class III phosphoinositide 3-kinase (PI3K-III, also known as PIK3C3) activity. Beclin-1 is a direct downstream target of ULK1, which regulates the lipid kinase activity of the PI3K-III catalytic unit vacuolar protein sorting34 (Vps34). Vps34 produces phosphatidylinositol 3-phosphate [PI (3-phosphate)], which enables the recruitment of many autophagic proteins involved in autophagosome nucleation. When autophagy is induced, the Vps34-Vps15-Beclin-1 complex can bind with different autophagy-related proteins, delivering autophagic signals, and promoting autophagy (McKnight and Zhenyu 2013). During the nucleation phase, the bilayer membrane structure of the phagophore wrap the molecules to be degraded to form a sequestering compartment. In this phase, the p62 acts as a bridging protein in the autophagy process, bringing the reduant. dysfunctional and damaged cellular components, like excess mitochondria and unfolded proteins to the phagophore, and then binds with microtubule-associated protein 1 light chain 3 [protein light chain3 (LC3)] (Chen, Lam et al., 2010; Tanaka, Jin et al., 2012; Ge, Wilz et al., 2015), which traps these cargo to be degradeds in the phagophore. The partition membrane continues to bend, elongate, and completely wraps around the cytoplasm to form a complete double-membrane autophagosome. Autophagosomes can fuse with late endosomes to form amphisomes and finally form autolysosomes by fusing with lysosome. Besides, autophagosomes also can directly fuse with lysosomes to form autolysosomes.

## The Regulatory Functions of Autophagy in Viral Infection

It has been previously reported that autophagy is not only a metabolic pathway in the organism to maintain homeostasis, but also it can effectively play antiviral functions as an irreplaceable component of the human immune defense system. After the virus invades into cells, the viral proteins, nucleic acids, and viral particles can be brought into the lysosome for degradation through autophagic signaling pathway, thus maintaining the health of the organisms (Levine 2005; Levine and Kroemer 2019; Yang and Klionsky 2020). Viruses are intracellular parasitic microorganisms with only one nucleic acid type. Autophagy may be triggered by any step in the life cycle of viruses, including viral adsorption and invasion nucleic acid replication and protein synthesis, and so on (Lünemann and Münz 2009; Tey and Khanna 2012; Perot, Ingersoll et al., 2013). Autophagy has antiviral or viral promotion functions during viral infection, depending on the virus, cell type, and cell environment (Jackson 2015; Pleet, Branscome et al., 2018). At present, it has been found that virus invasion induces autophagy in three ways. 1). Virus proteins can directly induce autophagy (Gannagé, Dormann et al., 2009; Londino, Lazrak et al., 2015). 2) Autophagy can be induced by virus ligand binding to the receptor (Joubert, Meiffren et al., 2009; Richetta, Grégoire et al., 2013). 3) Viruses-related endoplasmic reticulum stress induces autophagy. Viruses can encode a large number of viral proteins in infected cells. However, host cells cannot effectively modify these proteins in the endoplasmic reticulum. Those proteins cannot be correctly folded, resulting in protein accumulation in the endoplasmic reticulum, triggering endoplasmic reticulum stress, and activating autophagy in cells.

Autophagy can transport the virus in the cytoplasm to the lysosome by heterologous phagocytosis, directly degrade the virus, and transport viral pathogen-associated molecular patterns (PAMPs) to the intracellular receptors to activate the innate immunity. It can also present the virus antigen to the major histocompatibility complex (MHC) to activate the adaptive immunity and then trigger the function of resisting virus infection.

It is well known that innate immunity is the organism’s first-line defense against invasion by exogenous microorganisms. The PRRs (pattern recognition receptors) recognize and bind to specific molecular structures of pathogenic microorganisms and activate downstream signaling pathways to produce type I interferon (IFN), type III IFN, pro-inflammatory factors, and chemokines to establish the antiviral state (Kawai and Akira 2010; Stanifer, Pervolaraki et al., 2019).

Toll-like receptor (TLR) is a classical PRR that recognizes multiple viral nucleic acids. By recruiting adaptor molecule 1 (TRIF) or myeloid differentiation factor MyD88, the signal can be transmitted to the downstream NF-κB signaling pathway and synthesizes inflammatory factors and IFN regulatory factors (IRFs), promoting IFN production (Lee and Kim 2007; Negishi, Taniguchi et al., 2018). In this process, TLR can induce the interaction between TRIF or MyD88 and Beclin 1, by which the binding between Beclin 1 and Bcl⁃2 can thus induce autophagy (Zalckvar, Berissi et al., 2009; Kang, Zeh et al., 2011). On the other hand, autophagy can also be directly activated by pathogen-associated molecular patterns (PAMP) and transmit PAMP signals to TLRs to induce immune responses (Chen, Yang et al., 2012). Song J et al. found in human bronchial epithelial cells (16HBE) that autophagy could induce the increased replication of EV71 and CA16 (Song, Hu et al., 2018). Therefore, during the infection of a few viruses, autophagy can negatively regulate the TLR signaling pathway, thus contributing to the self-proliferation of the virus.

RIG-I-like receptors (RLRs) are localized in the cytoplasm and are mainly responsible for recognizing specific structures that are not present in normal cellular RNAs, such as the 5′ end triphosphate of viral dsRNA (Chiang, Davis et al., 2014). The RLRs sense the presence of the virus and interact with mitochondrial antiviral signals (MAVS) through the caspase recruitment domain (CARD), which in turn activates the regulatory factors IRF3 and IRF7 and NF-κB signaling pathways (Chiang, Davis et al., 2014). It was found that Sendai virus-infected cells could induce autophagy, but not in RIG-I-deficient cells (Lee, Ban et al., 2018), suggesting that RIG-I plays a role in virus-induced autophagy. The function of RIG-I is related to its molecular stability. In addition to ubiquitin-proteasome degradation, it also includes autophagic pathways. Leucine-rich Recombinant Protein 25 (LRRC25) can act as a secondary receptor to facilitate the recognition of ISG15-related RIG-I by SQSTM1/p62, mediating the degradation of RIG-I through selective autophagy (Du, Duan et al., 2018; Xu, Wan et al., 2018).

In addition, a few receptor molecules that sense DNA virus invasion in cells can also interact with the autophagic pathway. Cyclic GMP-AMP synthase (cGAS) can synthesize cyclic GMP -AMP (Cyclic GMP -AMP) after recognizing DNA viruses, followed by the STING factor binding to cGAMP to activate downstream related regulators and induce NF-kB activation. Among them, ERGIC containing STING can provide a membrane source for LC3 lipidation, thus promoting the clearance of cytoplasmic virus DNA by autophagy (Gui, Yang et al., 2019). On the other hand, during HSV-1 infection, Beclin 1 interacts with cGAS to inhibit cGAMP synthesis and IFN production and enhances autophagy-mediated degradation of cytoplasmic pathogen DNA to prevent excessive cGAS activation and sustained immune stimulation (Liang, Seo et al., 2014; Ni, Ma et al., 2020). Therefore, autophagy or autophagy-related proteins are not only regulated by signals such as cGAS-STING. However, they can also negatively regulate related signaling pathways.

Delivery of antigen is a transitional process during which natural immunity is the shift to adaptive immunity. In this process, antigen peptide fragments activate the major histocompatibility complex (MHC) in antigen-presenting cells (APCs), followed by antigen recognition by T cells in organisms. MHC mainly consists of two categories: MHC-I and MHC-II. MHC-I can be expressed in all nucleated cells. The viral protein can be delivered to CD8^+^ T cells through MHC-I-like molecules after degradation by the proteasome into immunogenic peptides, while autophagy limits MHC-I-like molecules by mediating their internalization and degradation (Choi, Bowman et al., 2018). The inactivation of the autophagy factors ATG5 and ATG7 in dendritic cells (DCs) can increase the expression of MHC-I on the cell surface, which makes the body induce a stronger immune response when infected with IAV and LCMV (Loi, Müller et al., 2016).

On the other hand, autophagosomes form constitutively in MHC-class II positive APCs such as dendritic cells, B cells, and epithelial cells, and APCs capture extracellular antigens and deliver them to autophagosomes. The autophagosomes fuse with MHC-II containing late nuclear endosomal compartments (MIIC) to deliver cytoplasmic antigen into MHC-II-like molecules, then delivered to CD4^+^ T cells (Dengjel, Schoor et al., 2005). Studies have shown that after HIV infection into DCs, cells can utilize LC3 fusion protein to target HIV antigens to autophagosomes specifically. In this way, the immune response of CD4^+^ T cells is effectively enhanced and amplified, thereby facilitating the restricted expression of MHC-II (Coulon, Richetta et al., 2016). In addition, autophagy can mediate the cross-antigen presentation of MHC-I and MHC-II types in APCs, facilitating information exchange between cells to generate effective immune responses against endogenous and exogenous antigens (English, Chemali et al., 2009). Autophagy also contributes to the developmental maturation of immune cells. The deficiency of autophagy-related genes, ATG5 or ATG7, in T cells can decrease the number of thymocytes and peripheral T cells. Besides, in ATG5-deficient T cells, the accumulation of damaged and aged mitochondria can cause cell proliferation problems and reduced cell viability (Stephenson, Miller et al., 2009). The mice with ATG7-deficient T cells fail to effectively establish CD8^+^ T cell responses to influenza and murine cytomegalovirus (MCMV) infection, suggesting that autophagy is essential for the survival of effector CD8^+^ T cells and immune memory formation (Puleston, Zhang et al., 2014; Xu, Araki et al., 2014).

## The Specific Functions of Several Classical Viruses

During the host infection, autophagy can degrade the virus and promote the innate and adaptive immunity of the host to inhibit viruses. However, it has been known that quite a few adaptive viruses have evolved a variety of biological mechanisms to regulate autophagy in the process of long-term co-existence with the host, such as using autophagy to inhibit cellular innate immunity to escape immune surveillance or by directly inhibiting the formation of autophagosomes, blocking the fusion of autophagosomes and lysosomes, and even using autophagosomes as a place for their replication to achieve the purpose of survival and reproduction. Studies have found that different viruses and different stages of infection have different strategies for regulating autophagy (Sir and Ou 2010; Abdoli, Alirezaei et al., 2018; Huang, Liu et al., 2020). The functions of autophagy in several viruses are described below.

Previous studies showed that x protein overexpression in hepatitis B virus (HBV) could significantly up-regulate Beclin 1 and promote autophagy levels in liver cancer cells (Tang, Da et al., 2009). Nevertheless, another study indicated that, by triggering endoplasmic reticulum stress, the small surface protein SHBs of HBV can effectively activate autophagy, and SHBs protein and autophagy protein LC3 co-localize and interact during HBV replication. The application of autophagy inhibitor 3—methyladenine (3-MA) to inhibit autophagy can significantly inhibit HBV production, and the application of rapamycin or starvation to induce autophagy can promote the production of HBV. The results suggested that autophagy was involved in HBV replication (Li, Liu et al., 2011).

Hepatitis C virus (HCV) is a single-stranded (+) RNA virus. Studies have shown that this HCV is also associated with autophagy. Its function may have two sides: HCV invading cells can cause endoplasmic reticulum stress, increasing autophagosome. However, the fusion of autophagosomes and lysosomes is inhibited. This incomplete autophagy mechanism allows HCV to use the autophagosomal membrane to replicate viral RNA, thus facilitating HCV replication (Shrivastava, Raychoudhuri et al., 2011). In contrast to this finding, other studies have found that the IFNbeta-induced endoplasmic reticulum transmembrane protein SCOTIN can recruit the NS5A protein from HCV to the autophagosome for degradation (Kim, Kim et al., 2016). In addition, IFN-λ1 can suppress the expression of ATG5 and GABARAP by inducing the expression of miR-181a and miR-214, thereby downregulating autophagy to inhibit HCV replication (Li, Li et al., 2017).

Avian influenza virus H5N1 infection can induce cells to produce inflammatory cytokines leading to body damage by producing ROS. H5N1 infection of A549 lung epithelial cells can induce the formation of obvious autophagosomes. Applying autophagy inhibitor 3-MA or knockdown of autophagy gene Atg5 by siRNA can alleviate H5N1-induced acute lung injury. Further studies have found that H5N1 can mediate lung injury by inhibiting Akt/TSC2/mTOR pathway to activate autophagy (Imai, Kuba et al., 2008). Therefore, targeting the autophagy pathway could be a promising method for the medical treatment of lung inflammation caused by the H5N1 virus.

The human immunodeficiency virus (HIV) can manipulate the autophagy process by interacting with different autophagy factors. In the early stage of autophagy, HIV Gag protein binds and interacts with LC3, thereby promoting the processing of Gag protein and the maturation and packaging of viral particles for HIV replication. When autophagy enters the mature stage, HIV Nef protein plays an anti-autophagy maturation role by interacting with autophagy protein Beclin-1, thereby protecting HIV from degradation and promoting the release of mature virus particles. Therefore, HIV can promote HIV survival and replication by interfering with different stages of autophagy (Kyei, Dinkins et al., 2009; Leymarie, Lepont et al., 2017).

Human Bocavirus 1 (HBoV1) is a parvovirus, which can cause serious respiratory diseases in children. NP1 protein of HBoV1 can up-regulate Beclin1 and LC3II in A549 cells but down-regulate the expression of p62 and HMGB1 and inhibit cell survival and migration. In addition, it is also found that the STAT3 signaling pathway activates NP1-induced autophagy in A549 cells. The above results show that NP1 of HBoV1 induces autophagy in A549 cells by activating STAT3 signaling pathway phosphorylation and inhibits the viability of A549 cells. However, the mechanism of NP1-induced autophagy remains unclear (Kim, Lee et al., 2010; Lennemann and Coyne 2015; Mancias and Kimmelman 2016; Padman, Nguyen et al., 2017).

## Autophagy and SARS-CoV-2

A new coronavirus was discovered in late 2019 (Severe acute respiratory syndrome coronavirus 2. SARS-CoV-2), which spread rapidly in a short period and attracted worldwide attention. With the COVID-19 pandemic caused by the SARS-Cov-2, as of September 9, 2021, more than 222,136,320 cases were reported worldwide, including 4,590,565 death reports (Data from coronavirus resource center of Johns Hopkins University). Due to the epidemic’s severity, SARS-CoV-2 is currently one of the most important viruses in the academic community. Developing a specific drug or vaccine for SARS⁃CoV⁃2 has become a hot research topic (Weston, Coleman et al., 2020). Combing with the findings on other coronaviruses and the previous clinical survey showed that lots of medicines approved by the US Food and Drug Administration (FDA) can effectively regulate autophagic pathway (Bello-Perez, Sola et al., 2020; Shojaei, Suresh et al., 2020). This viewpoint suggests that the accumulation of autophagsosomes caused by interfering autophagic pathway may be a potential target for controlling SARS-CoV-2 by inducing apoptosis of infected cells. As proof of concept, both Chloroquine (CQ) and Hydroxychloroquine (HCQ) (HCQ) are two of the drugs of interest for clinical evaluation (Elavarasi, Prasad et al., 2020; Kumar, Jain et al., 2021; Singh, Ryan et al., 2021). They can inhibit the autophagic process by increasing the pH of acidic endosomes/lysosomes. In contrast to this point, other study found that through inhibiting the function of autophagy resisting pathogens, SARS-CoV-2 can survive and replicate in cells without protection. Therefore, increasing the autophagy flux level is also the way to treat SARS-CoV-2 (Miao, Zhao et al., 2021). Hannan et al. mentioned that intermittent fasting could be a potential approach to treat the infection SARS-CoV-2 by the activation of autophagy (Hannan, Rahman et al., 2020). These opinions suggest that autophagy regulation is indispensable in the fighting against SARS-CoV-2 infection.

## Summary and Perspectives

Autophagy, as a conservatively evolved intracellular degradation pathway, is a multi-step and complex process. Autophagy can deliver the virus PAMPs to pattern recognition receptors to activate innate immunity. Additionally, it can present viral antigens processing to MHC molecules to activate adaptive immunity in organisms. It can also transport the virus in the cytoplasm to the lysosome by heterologous phagocytosis, which plays a role in resisting virus infection. On the other hand, viruses can promote their infection by inhibiting autophagy, interacting with autophagy proteins, and even using autophagy as a replication site. Therefore, an in-depth study of the relationship between autophagy and viral infection is helpful to provide new ideas and targets for controlling viral infection.

The COVID-19 epidemic has not yet been truly resolved, and clinically effective drugs and vaccines available are still under investigation. Autophagy may be a good potential regulatory target for the treatment of the COVID-19 target. In addition, it should be noted that the regulatory function of autophagy in viral infection varies greatly depending on the virus types and the environment in which it is located. It is important to remind us to consider effectively utilizing the autophagy process as a therapeutic pathway to treat diseases caused by viruses.
